# The impact of sound intervention on sleep quality and stress levels in college students: a randomized controlled trial

**DOI:** 10.3389/fpsyg.2026.1859138

**Published:** 2026-06-17

**Authors:** Hengfei Hou, Jian Wang, Xiaodong Yue

**Affiliations:** 1 Henan Forestry Vocational College, Luoyang, Henan, China; 2 School of Psychology, Capital Normal University, Beijing, China

**Keywords:** binaural beats, pink noise, salivary cortisol, sleep quality, stress

## Abstract

**Introduction:**

To assess the effectiveness of interventions combining pink noise or binaural beats with pure music in improving sleep quality and reducing perceived stress among college students, and to preliminarily investigate their effects on morning salivary cortisol levels.

**Methods:**

Using a three-group randomized controlled design, 66 college students with poor sleep quality (PSQI score >7) were randomly assigned to a pink noise + pure music group, a binaural beats + pure music group, or a control group. The intervention group received a 30-min audio intervention at bedtime for 4 consecutive weeks, 5 nights per week. Morning salivary cortisol concentrations were measured at baseline, week 2, week 4, and the week-6 follow-up. Perceived stress and sleep quality were assessed using the PSS and PSQI, respectively.

**Results:**

Generalized estimating equation analyses revealed significant time, group, and time-by-group interaction effects for both PSS and PSQI scores (*p* < 0.05). The binaural beats + pure music group was more effective at reducing stress. Morning salivary cortisol concentrations showed only a significant main effect of time (*p* < 0.05), with no significant group differences.

**Discussion:**

A 4-week bedtime audio intervention effectively improved sleep quality and reduced perceived stress. Binaural beats combined with pure music appeared to be more effective in reducing perceived stress, but this benefit was not reflected in peak salivary cortisol levels measured 30 min after awakening. However, cortisol measurements obtained at a single daily time point are insufficient to infer a short-term modulatory effect of the intervention on basal hypothalamic–pituitary–adrenal (HPA) axis activity.

## 1 Introduction

In recent years, declining sleep quality among college students has become a high-profile public health issue, attracting widespread attention in higher education and mental health. Sleep disorders in this group are usually the result of intertwined environmental and psychological factors. In terms of environment, uncontrollable noise in dormitories is the primary factor affecting sleep. In particular, persistent noise stemming from differences in roommates’ routines can directly lead to delayed sleep onset and sleep disruption (Meng et al., 2020). Meanwhile, at the psychological level, common stressors such as academic competition, interpersonal relationships and future career development can significantly increase an individual’s anxiety level, which can further aggravate the aforementioned sleep disturbances. It is worth noting that the relationship between stress and sleep is bidirectional: stress increases cognitive arousal and emotional activation before bedtime, which in turn leads to difficulty falling asleep and reduced sleep. Reduced sleep, in turn, weakens the prefrontal cortex’s ability to regulate emotions. The negative emotions and stress levels on the next day will increase, further intensifying the overall sense of stress (Yao et al., 2025). According to this framework, a great deal of psychoneuroendocrine research has shown that cortisol, cortisol, the primary end product of the hypothalamic–pituitary–adrenal (HPA) axis, is intimately associated with stress and sleep (Cagnacci et al., 2025; Dosseva-Panova et al., 2025; Hertenstein et al., 2025; Martinez-Moral & Kannan, 2026). Salivary cortisol is an ideal non-invasive biomarker commonly used in scientific research. Studies have shown that salivary cortisol concentrations correlate well with plasma cortisol concentrations and can effectively reflect cortisol levels in the body (El-Farhan et al., 2017; Elder et al., 2014; Poll et al., 2007). Studies have shown that elevated bedtime cortisol levels lead to three common effects: shorter total sleep duration, reduced sleep efficiency, and prolonged sleep latency. Chronic poor sleep quality results in impaired basal activity of the HPA axis, blunted circadian rhythms, and a flattened cortisol secretion profile (Liu, 2024). The key to solving the problem lies in finding effective interventions that both relieve psychological stress and minimize external environmental disturbances.

Traditional pharmacological interventions (e.g., sedative-hypnotic drugs), although rapidly effective, are prone to drug resistance, addiction, and next-day residual effects, and are not suitable as routine interventions for college students (Sateia et al., 2017). Therefore, it is particularly urgent to explore a simple, non-invasive, low-cost and highly acceptable non-pharmacological intervention. In this context, pre-bedtime audio intervention has advantages due to its ease of use and the dual effects of physical masking and neuromodulation (Hu et al., 2023). Currently, the three most commonly used audio intervention methods are white noise, pink noise, and binaural beats (Gantt, 2023). Although white noise, with its uniform distribution of acoustic energy across the entire frequency range, can effectively mask disruptive ambient noise, its monotonous acoustic characteristics often give rise to significant individual differences—some people find it soothing, whilst others find it unpleasant or even repulsive (Wu, 2025). Pink noise, on the other hand, is concentrated in the lower frequencies and gradually attenuates at higher frequencies; it is less jarring to the ear. As its spectral characteristics more closely resemble natural sounds and are more in tune with the brain’s natural brainwave frequencies, it is generally more pleasant to listen to when masking environmental noise (Capezuti et al., 2022). When two pure tones with slightly different frequencies are presented to each ear independently, a third binaural beat equal to the frequency difference is perceived (Bang et al., 2019). Certain audio frequencies can entrain neural oscillations, potentially resulting in brainwave states associated with sleep and relaxation. The project was originally approved under the title “The Effect of White Noise on Sleep Quality in University Students: A Controlled Experimental Study Based on Universities in Henan Province”, which aimed to investigate the sleep-enhancing effects of white noise. During the initial phase of the project, we conducted a pilot study, in which some participants reported a poor subjective experience and found it difficult to tolerate over the long term. Conversely, when white noise was replaced with pink noise and binaural beats, participants reported a significant increase in acceptance. Based on the results of the pilot study, and in light of previous literature highlighting the advantages of pink noise and binaural beats in terms of acoustic comfort and neuromodulation, this study adjusted the intervention material from white noise to a combination of pink noise and binaural beats in the formal trial, with the aim of enhancing the ecological validity of the intervention and improving participant compliance.

Although existing evidence suggests that both pink noise and binaural beats can significantly improve sleep quality (Abeln et al., 2014; Elnazer, 2026), and binaural beats additionally show positive effects on mood regulation, stress and pain reduction, and enhancement of memory and attention (Engelbregt et al., 2021; Lee et al., 2022; Yang Y. et al., 2025). However, since listening to pink noise or binaural beats alone may not be acceptable to everyone, pure music is usually added to enhance both acceptability and the desired effect (Lee et al., 2019). Nevertheless, there are two limitations in existing studies: one, the evidence for directly comparing the effects of pink noise and binaural beats is not yet sufficient; and two, the effects of interventions targeting college student populations in real-life scenarios remain to be empirically evaluated. Therefore, the present study proposes a 4-week bedtime audio intervention in a randomized controlled trial in a natural life setting for college students. A control group, a pink-noise group, and a binaural-beats group were established. The effects of the two audio interventions on sleep quality and perceived stress were systematically evaluated using the Perceived Stress Scale (PSS-10), the Pittsburgh Sleep Quality Index (PSQI), and morning salivary cortisol measurements, in an attempt to provide empirical evidence for the development of an intervention that is suitable for the campus environment and easy to be replicated.

## 2 Methods

### 2.1 Participants

**Figure 1:**
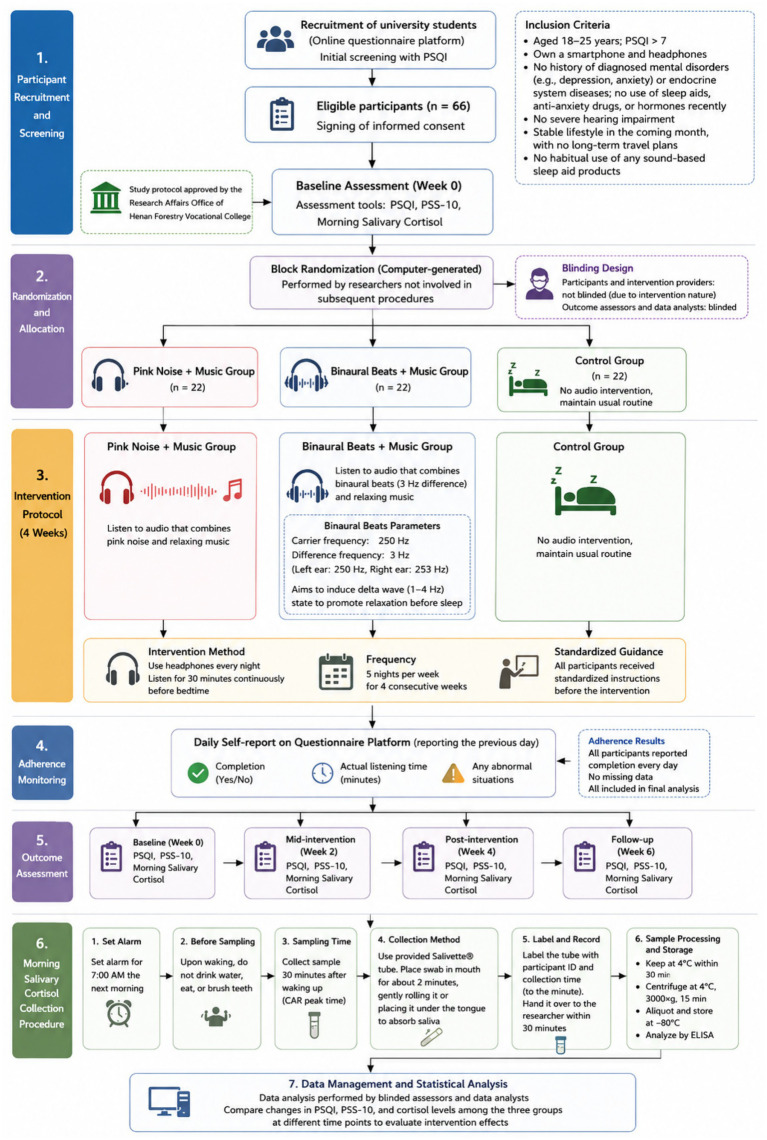
The CONSORT study flow diagram.

The sample size was estimated using GPower 3.1 software, and there is a lack of exact *a priori* effect sizes for studies on sound interventions aimed at improving sleep quality and stress levels in college students. Considering the cost of the experiment and the need for both statistical validity and scientific rationality. Referring to Cohen’s criteria for moderate effects, the effect size was set at *f* = 0.25, *α* = 0.05, and the test power (1-*β*) = 0.95 (Cohen J., 1988). Subjects were randomly assigned to three groups: pink noise + pure music group, binaural beats + pure music group, and control group. Four repeated measurements were taken at baseline, the second week of the intervention, the fourth week of the intervention, and the sixth week of the follow-up visit. Substituting the above parameters into the software calculation yielded a total sample size of 57 required for the three groups. After accounting for potential dropout, a total of 66 subjects who met the criteria were included, 22 in each group. The PSQI was first used to screen participants using the Wenjuanxing platform, a well-known Chinese online survey platform. The requirements for inclusion included being between the ages of 18 and 25, having a PSQI score greater than 7, owning a smartphone and headphones, not having a diagnosis of endocrine or psychiatric disorders (such as depression or anxiety disorders), not having recently used sleep aids, anxiolytics, or hormone medications, not having severe hearing impairment, leading a relatively stable lifestyle with no plans to travel long distances in the near future, and not regularly using any sound-assisted sleep products. The institutional office responsible for research and international relations reviewed and approved the study protocol. Every procedure was carried out strictly in compliance with the applicable ethical guidelines. Prior to participation, each subject provided written informed consent after being fully informed of their rights and the study’s methods. The flowchart for this study is shown in Figure 1.

### 2.2 Intervention and study design

#### 2.2.1 Randomization and blinding

This study used a single-masked, evaluator-blinded, three-group parallel randomized controlled design with a 4-week intervention period and a follow-up visit at week 6. A computer-generated block randomization sequence was used by researchers not involved in the follow-up process to assign subjects to the pink noise + pure music group, the binaural beats + pure music group, and the control group, and grouping information was sealed. Due to the nature of the intervention content, subjects and intervention implementers could not be blinded, but outcome assessors and data analysts were blinded to the groupings.

#### 2.2.2 Intervention program

All intervention audio was generated using MATLAB 2021a and professionally calibrated. Subjects were required to listen to the specified audio continuously through headphones for 30 min before bedtime every night, 5 days a week, for 4 weeks. They received uniform instructions before the start of the intervention. Pink Noise + pure music group: Listening to a mixture of pink noise and pure music. Binaural beats + pure music group: listening to audio containing specific frequencies of binaural beats mixed with pure music. Control group: no audio interventions, maintain routine.

#### 2.2.3 Sound material

The pure music added to both intervention groups was the same; the tempo of sleep-aid music is usually kept between 60–80 BPM (Li et al., 2025), and River Flows in You and Canon, which are well-known and fit this range, were selected for this study. Binaural beats with pink noise and audio mixes were generated by MATLAB 2021a, where binaural beats were generated using 250 HZ as the carrier frequency with a 3 Hz differential frequency superimposed, i.e., the left and right ears were presented with a sine wave of 250 Hz and 253 Hz, respectively, in a configuration designed to induce *δ*-wave (1–4 Hz) states that promote relaxation at bedtime (Li et al., 2025).

#### 2.2.4 Adherence tracking

The intervention was conducted in a dormitory setting, and compliance monitoring was limited to the two audio intervention groups (22 participants in each, totalling 44). Compliance was self-reported via a questionnaire the following day, recording whether the listening session had been completed, the duration of listening, and any irregularities. The control group was not included in this analysis. It should be noted that compliance assessment relied on subjective reporting and was not objectively verified via headphones or software back-end systems; whilst convenient, this approach carries the potential for recall bias and social desirability bias (see the Limitations section for details).

The experiment required participants to listen for 5 days a week, for 4 consecutive weeks (totalling 20 sessions). Each session had to be completed in full for 30 min to be counted as a valid task; if the actual listening duration was less than 30 min but at least 25 min (i.e., a shortfall of 5 min or less), although not counted as a valid task, it was still considered as “intervention completed” and retained in the dataset. Given the difficulty of fully standardising the dormitory environment, as long as participants reported having completed 20 sessions of the intervention—achieving a 90% validity rate (i.e., at least 18 sessions)—they were not excluded, thereby avoiding the loss of valid samples due to a shortfall of just one or two sessions.

Self-reported results indicate a high level of compliance, of the 44 participants in the intervention group, 37 (84.1%) strictly completed all 20 listening tasks, each lasting 30 min; 7 (15.9%) achieved a 90% validity rate (3 completed 19 sessions and 4 completed 18 sessions), and no participant reported failing to listen at all. All 66 participants (including those in the control group) completed data collection as scheduled at all four time points—baseline, week 2 of the intervention, at the end of week 4 of the intervention, and at the week 6 follow-up period—with no attrition. However, it should be noted that this high retention rate is a result of specific circumstances, and its generalisability to open communities may be limited.

### 2.3 Measures

#### 2.3.1 Pittsburgh sleep quality index (PSQI)

There are eighteen self-rated items in the Pittsburgh Sleep Quality Index (PSQI). Subjective sleep quality, sleep latency, sleep duration, sleep efficiency, sleep disruptions, use of sleep medication, and daytime dysfunction are the seven variables for which it may provide scores (Liu et al., 1996). A score higher than 7 indicates poor sleep quality on the PSQI, which has a total range of 0 to 21. The PSQI’s Cronbach’s alpha coefficient in this investigation was 0.83.

#### 2.3.2 Perceived stress scale (PSS-10)

There are 10 items, 6 positively scored items measuring the crisis perception factor (items 1, 2, 3, 6, 9, and 10), and 4 negatively scored items measuring the coping ability perception factor (items 4, 5, 7, and 8), and the sum of the two factors is the total score (Chen et al., 2021; Cohen S., 1988). The total score ranges from 0–40, with the higher the score, the higher the perceived stress. The Cronbach’s alpha coefficient for this scale in this study was 0.84.

#### 2.3.3 Morning salivary cortisol

The saliva sample collection strictly followed a standardized protocol. All participants were instructed to set a unified wake-up alarm for 7:00 a.m. Upon waking, they remained at rest, refraining from drinking, eating, and brushing teeth. Samples were self-collected 30 min after waking (approximating the peak of the cortisol awakening response) using Salivette® saliva collection tubes (Sarstedt, Germany). Participants were instructed to avoid touching the swab head with their hands, to gently roll the swab in the mouth or place it under the tongue for approximately 2 min. Saturated swabs were returned to the tubes, which were immediately labeled with participant ID and collection time (to the minute). Samples were submitted to an on-site researcher within 30 min, temporarily stored at 4 °C, centrifuged (4 °C, 3000 × *g*, 15 min) on the same day, and the supernatant was aliquoted and stored at −80 °C until batch analysis using a competitive enzyme-linked immunosorbent assay (ELISA).

### 2.4 Statistical analysis

Data were analyzed using SPSS 31.0. As continuous variables violated the normality assumption (Shapiro–Wilk test, *p* < 0.05), baseline between-group comparisons were conducted using the Kruskal-Wallis H test for continuous variables and the chi-square test for categorical variables—the primary analysis employed Generalized Estimating Equations (GEE). Separate GEE models were constructed with salivary cortisol concentration, PSS score, and PSQI score as dependent variables. Time, group, and their interaction were included as factors. Based on the distribution characteristics, a Gamma distribution with a log link function and an exchangeable working correlation matrix was specified. If a significant interaction effect was found, simple effect analyses were conducted, with all pairwise comparisons adjusted using the Bonferroni method. The significance level was set at *α* = 0.05 (two-tailed).

## 3 Results

### 3.1 Baseline characteristics

No significant differences were found among the three groups at baseline in terms of demographic characteristics (gender, major, grade), salivary cortisol concentration, PSS scores, or PSQI scores (*p* > 0.05), indicating successful randomization and good comparability (Table 1).

**Table 1:** Baseline comparison of the three groups of subjects.

Variable	Pink noise + pure music group (*n* = 22)	Binaural beats + pure music group (*n* = 22)	Control group (*n* = 22)	H/Wald χ^2^	*p*
Gender				0.496	0.78
Male	8 (36.4%)	10 (45.5%)	10 (45.5%)		
Female	14 (63.6%)	12 (54.5%)	12 (54.5%)		
Major				0.901	0.637
Engineering	8 (36.4%)	7 (31.8%)	10 (45.5%)		
Agriculture	14 (63.6%)	15 (68.2%)	12 (54.5%)		
Grade					
Freshman	11 (50%)	8 (36.4%)	11 (50%)	2.156	0.707
Sophomore	9 (40.9%)	11 (50%)	7 (31.8%)		
Junior	2 (9.1%)	3 (13.6%)	4 (18.2%)		
Baseline cortisol (ng/mL)	18.32 (15.5, 20.1)	17.78 (11.5, 22.9)	16.5 (11, 25.25)	0.116	0.943
Baseline PSS	26 (22, 28)	23 (22, 28)	24 (22, 26)	2.654	0.265
Baseline PSQI	9.5 (9, 11)	9.5 (9, 11)	10 (9, 11)	0.17	0.918

### 3.2 Changes in salivary cortisol

The median salivary cortisol concentrations across the four time points for the three groups, along with the results of the Generalised Estimating Equations (GEE) analysis, are presented in Table 2. The GEE analysis revealed a significant main effect of time (Wald χ^2^ = 9.974, df = 3, *p* = 0.019), indicating that cortisol concentrations showed an overall downward trend as the study progressed; the main effect of group was not significant (Wald χ^2^ = 2.048, df = 2, *p* = 0.359), suggesting that, when time was not taken into account, there was no statistically significant difference in overall concentrations among the three groups; The time × group interaction effect was also not significant (Wald χ^2^ = 3.819, df = 6, *p* = 0.701), indicating that the overall trends in changes over time across the three groups did not differ markedly.

**Table 2:** Results of generalized estimating equation analysis of salivary cortisol concentrations in three groups of subjects at four time points.

Variable	Pink noise + pure music group (*n* = 22)	Binaural beats + pure music group (*n* = 22)	Control group (*n* = 22)	Wald *χ^2^*(df)	*p*
Baseline	18.32 (15.5, 20.1)	17.78 (11.5, 22.9)	16.5 (11.0, 25.25)	0.14 (2)	0.932
Week 2	15.87 (8.7, 18.5)	16.01 (14.59, 18.09)	12.0 (7.75, 22.5)	2.881 (2)	0.237
Week 4	15.0 (13.9, 16.5)	16.0 (15.56, 17.13)	14.0 (12.0, 20.25)	6.019 (2)	0.049
Week 6 follow-up	14.59 (13.46, 16.59)	16.0 (14.95, 18.0)	13.5 (8.75, 23.0)	5.506 (2)	0.064
Wald χ^2^	5.282	0.835	9.224		
*p*	0.152	0.841	0.026		
Time effect				9.974 (3)	0.019
Group effect				2.048 (2)	0.359
Interaction effect				3.819 (6)	0.701

Data are presented as median (interquartile range).

### 3.3 Changes in perceived stress scale (PSS) scores

**Figure 2:**
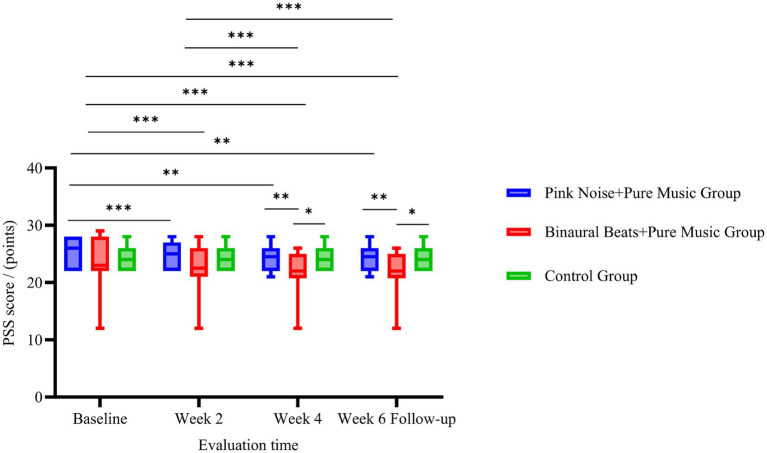
Changes in PSS of the three groups at four time points.

The median PSS scores for the three groups at the four time points and the results of the generalised estimating equation analysis are shown in Table 3. The GEE analysis revealed that the main effect of time was significant (Wald χ^2^ = 44.034, df = 3, *p* < 0.001), the main effect of group was significant (Wald χ^2^ = 6.323, df = 2, *p* = 0.042), and the time × group interaction effect was also significant (Wald χ^2^ = 44.388, df = 5, *p* < 0.001), indicating that the trends in PSS scores over time across the three groups differed statistically, warranting further analysis of simple effects (See Tables 4, 5; Figure 2).

**Table 3:** Results of generalized estimating equation analysis of PSS scores of three groups of.

Variable	Pink noise + pure music group (*n* = 22)	Binaural beats + pure music group (*n* = 22)	Control group (*n* = 22)	Wald *χ^2^*(df)	*p*
Baseline	26 (22, 28)	23 (22, 28)	24 (22, 26)	3.974 (2)	0.137
Week 2	25 (22, 27)^a^	22.5 (21, 26)^a^	24 (22, 26)	4.793 (2)	0.091
Week 4	24.5 (22, 26)^a^	22 (20.75, 25)^a,b^▲	24 (22, 26)▼	11.19 (2)	0.004
Week 6 Follow-up	24.5 (22, 26)^a^	22 (20.75, 25)^a,b^▲	24 (22, 26)▼	11.167 (2)	0.004
Wald χ^2^ (df)	22.000 (2)	41.003 (2)	1.225 (3)		
*p*	<0.001	<0.001	0.747		
Time effect				44.034 (3)	<0.001
Group effect				6.323 (2)	0.042
Interaction effect				44.388 (5)	<0.001

Data are presented as median (interquartile range), ▲ *p* < 0.05 compared with the pink noise + pure music group, ▼ *p* < 0.05 compared with the binaural beats + pure music group, *^a^p* < 0.05 compared with Baseline, *^b^p* < 0.05 compared with Week 2.

**Table 4:** GEE-estimated between-group mean difference and 95% CI (PSS).

Time	Group	MD (95% CI)	Adjusted *p*-value
Week 4	Pink noise + pure music group vs. binaural beats + pure music group	2.55 (0.69, 4.40)	0.003
	Pink noise + pure music group vs. control group	0.45 (−0.99, 1.90)	1.000
	Binaural beats + pure music group vs. control group	−2.09 (−3.92, −0.26)	0.018
Week 6	Pink noise + pure music group vs. binaural beats + pure music group	2.55 (0.69, 4.40)	0.003
	Pink noise + pure music group vs. control group	0.45 (−1.03, 1.94)	1.000
	Binaural beats + pure music group vs. control group	−2.09 (−3.94, −0.24)	0.021

**Table 5:** GEE-estimated within-group mean difference and 95% CI (PSS).

Time	Group	MD (95% CI)	Adjusted *p*-value
Pink noise + pure music group	Baseline vs. Week 2	0.50 (0.22, 0.78)	<0.001
	Baseline vs. Week 4	0.73 (0.19, 1.27)	0.002
	Baseline vs. Week 6 follow-up	0.73 (0.19, 1.27)	0.002
	Week 2 vs. Week 4	0.23 (−0.15, 0.60)	0.668
	Week 2 vs. Week 6 follow-up	0.23 (−0.15, 0.60)	0.668
	Week4 vs. Week 6 follow-up	0 (0, 0)	–
Binaural beats + pure music group	Baseline vs. Week 2	1.00 (0.58, 1.42)	<0.001
	Baseline vs. Week 4	1.95 (1.10, 2.81)	<0.001
	Baseline vs. Week 6 follow-up	1.95 (1.10, 2.81)	<0.001
	Week 2 vs. Week 4	0.95 (0.43, 1.48)	<0.001
	Week 2 vs. Week 6 follow-up	0.95 (0.43, 1.48)	<0.001
	Week4 vs. Week 6 follow-up	0 (0, 0)	–
Control group	Baseline vs. Week 2	−0.14 (−0.49, 0.22)	1.000
	Baseline vs. Week 4	−0.09 (−0.62, 0.44)	1.000
	Baseline vs. Week 6 follow-up	−0.09 (−0.50, 0.32)	1.000
	Week 2 vs. Week 4	0.05 (−0.45, 0.54)	1.000
	Week 2 vs. Week 6 follow-up	0.05 (−0.45, 0.54)	1.000
	Week4 vs. Week 6 follow-up	0.00 (−0.59, 0.59)	1.000

Intergroup comparisons (Table 4) showed that at the 4-week and 6-week follow-ups, the PSS scores for the Binaural Beats plus pure music group were significantly lower than those for the Pink Noise plus pure music group, with a mean difference of 2.55 (95% CI: 0.69–4.40) in both cases, and a corrected *p*-value of 0.003 in both instances. At the same time, the Binaural Beats group also scored significantly lower than the control group: at week 4, the mean difference was −2.09 (95% CI: −3.92 to −0.26), with a corrected *p* = 0.018; and at week 6, the mean difference was −2.09 (95% CI: −3.94 to −0.24), with a corrected p-value of 0.021. The differences between the Pink Noise combined with pure music group and the control group at all time points were not statistically significant (all corrected *p*-values >0.05).

Intra-group comparisons (Table 5) showed that PSS scores in the Pink Noise combined with pure music group decreased from baseline immediately following the intervention. At Week 2 (mean difference 0.50, 95% CI: 0.22–0.78), Week 4 and Week 6 (mean difference 0.73 at both time points, 95% CI: 0.19–1.27) were significantly lower than baseline (*p* ≤ 0.002), but there were no further significant changes between time points after week 2 (*p* > 0.05). The downward trend was more pronounced in the Binaural Beats combined with pure music group: a reduction of 1.00 (95% CI: 0.58–1.42) from baseline was observed as early as week 2, and by weeks 4 and 6, the reduction from baseline had reached 1.95 (95% CI: 1.10–2.81), both *p* < 0.001; furthermore, the scores at weeks 4 and 6 were significantly lower than those at week 2 (mean difference 0.95, 95% CI: 0.43–1.48, *p* < 0.001), indicating that the group’s perceived stress continued to improve during the intervention period, entering a plateau phase after week 4. Pairwise comparisons between time points in the control group did not reach statistical significance (*p* > 0.05), and the overall time effect was also not significant (Wald χ^2^ = 1.225, *p* = 0.747).

In summary, both music intervention protocols were effective in reducing stress perception levels among university students. The combination of Binaural Beats and pure music yielded superior results to the combination of Pink Noise and pure music. This advantage was maintained at both the 4-week and 6-week follow-ups, and the stress relief in the Binaural Beats group exhibited a pattern of sustained decline followed by stabilization. No meaningful changes in stress levels were observed in the control group throughout the study period.

### 3.4 Changes in Pittsburgh sleep quality index (PSQI) scores

**Figure 3:**
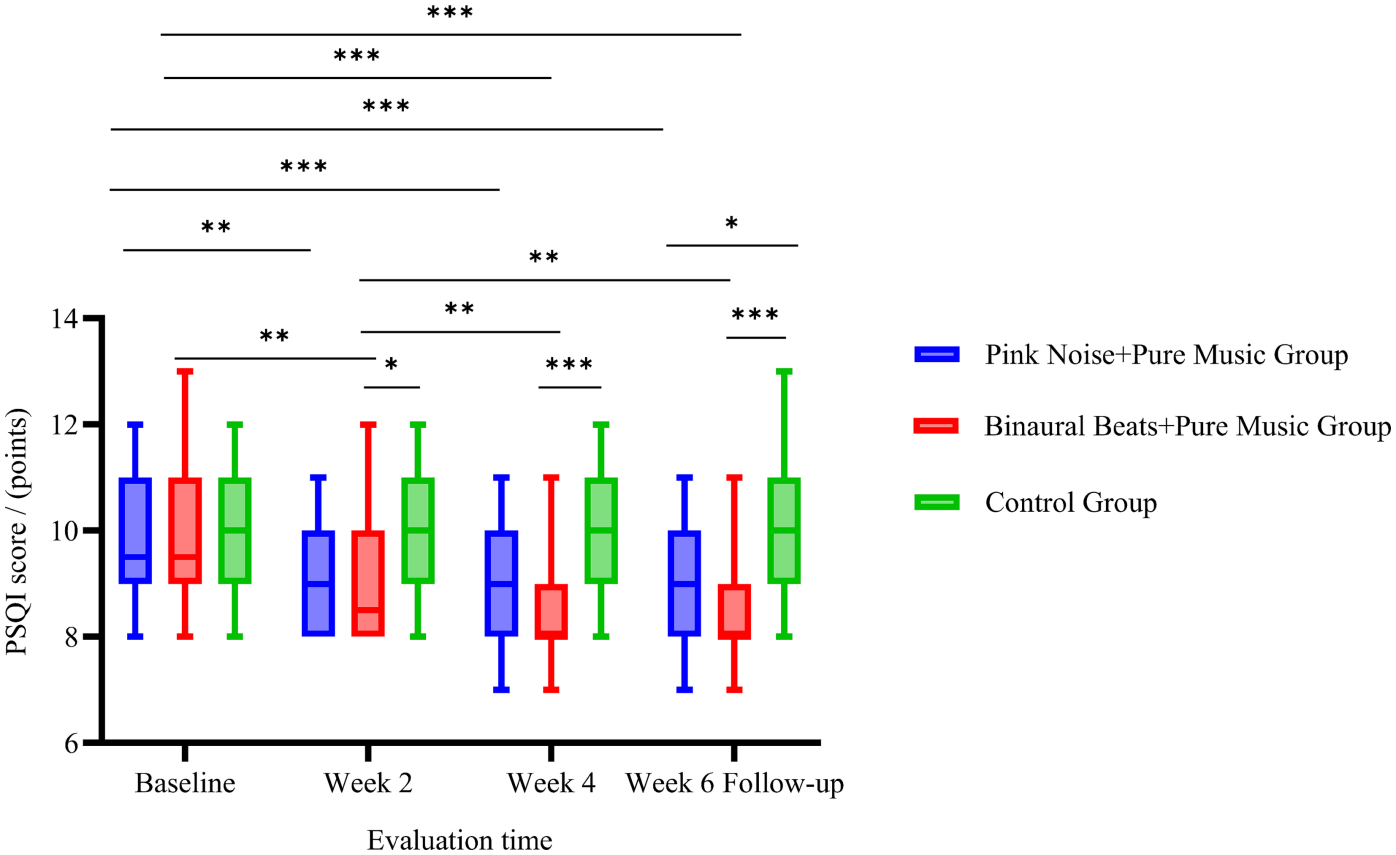
Changes in PSQI of the three groups at four time points.

The median PSQI scores for the three groups at the four time points and the results of the generalized estimating equation analysis are shown in Table 6. The GEE analysis revealed that the main effect of time was significant (Wald χ^2^ = 111.388, df = 3, *p* < 0.001), the main effect of group was significant (Wald χ^2^ = 7.453, df = 2, *p* = 0.024), and the time × group interaction effect was also significant (Wald χ^2^ = 165.128, df = 6, *p* < 0.001), indicating that the trends in PSQI scores over time differed statistically across the three groups, warranting further analysis of simple effects (See Tables 7, 8; Figure 3).

**Table 6:** Results of generalized estimating equation analysis of PSQI scores of three groups of subjects at four time points.

Variable	Pink noise + pure music group (*n* = 22)	Binaural beats + pure music group (*n* = 22)	Control group (*n* = 22)	Wald *χ^2^*(df)	*p*
Baseline	9.5 (9, 11)	9.5 (9, 11)	10 (9, 11)	0.163(2)	0.922
Week 2	9 (8, 10)^a^	8.5 (8, 10)^a^	10 (9, 11)^▼^	7.687(2)	0.021
Week 4	9 (8, 10)^a^	8 (8, 9)^a,b^	10 (9, 11)^▼^	15.407(2)	<0.001
Week 6 follow-up	9 (8, 10)^a^	8 (8, 9)^a,b^	10 (9, 11)^▲▼^	16.843(2)	<0.001
Wald χ^2^ (df)	23.594 (3)	139.333 (2)	2.200 (1)		
*p*	<0.001	<0.001	0.138		
Time effect				111.388(3)	<0.001
Group effect				7.453(2)	0.024
Interaction effect				165.128(6)	<0.001

Data are presented as median (interquartile range), ▲ *p* < 0.05 compared with the Pink Noise + Pure Music group, ▼ *p* < 0.05 compared with the Binaural Beats + Pure Music group, ^a^*p* < 0.05 compared with Baseline, ^b^*p* < 0.05 compared with Week 2.

**Table 7:** GEE-estimated between-group mean difference and 95% CI (PSQI).

Time	Group	MD (95% CI)	Adjusted *p*-value
Week2	Pink noise + pure music group vs. Binaural beats + pure music group	0.36 (−0.35, 1.07)	0.659
	Pink noise + pure music group vs. Control group	−0.55 (−1.28, 0.19)	0.228
	Binaural beats + pure music group vs. Control group	−0.91 (−1.70, −0.12)	0.017
Week 4	Pink noise + pure music group vs. Binaural beats + pure music group	0.50 (−0.24, 1.24)	0.319
	Pink noise + pure music group vs. Control group	−0.73 (−1.53, 0.08)	0.092
	Binaural beats + pure music group vs. Control group	−1.23 (−1.98, −0.48)	<0.001
Week6	Pink noise + pure music group vs. Binaural beats + pure music group	0.45 (−0.31, 1.22)	0.465
	Pink noise + pure music group vs. Control group	−0.86 (−1.71, −0.02)	0.044
	Binaural beats + pure music group vs. Control group	−1.32 (−2.09, −0.55)	<0.001

**Table 8:** GEE-estimated within-group mean difference and 95% CI (PSQI).

Time	Group	MD (95% CI)	Adjusted *p*-value
Pink noise + pure music group	Baseline vs. Week 2	0.41 (0.13, 0.69)	0.001
	Baseline vs. Week 4	0.59 (0.27, 0.92)	<0.001
	Baseline vs. Week 6 follow-up	0.64 (0.27, 1.00)	<0.001
	Week 2 vs. Week 4	0.18 (−0.04, 0.40)	0.162
	Week 2 vs. Week 6 follow-up	0.23 (−0.06, 0.52)	0.234
	Week4 vs. Week 6 follow-up	0.05 (−0.07, 0.16)	1.000
Binaural beats + pure music group	Baseline vs. Week 2	0.86 (0.67, 1.06)	<0.001
	Baseline vs. Week 4	1.18 (0.82, 1.55)	<0.001
	Baseline vs. Week 6 follow-up	1.18 (0.82, 1.55)	<0.001
	Week 2 vs. Week 4	0.32 (0.06, 0.58)	0.008
	Week 2 vs. Week 6 follow-up	0.32 (0.06, 0.58)	0.008
	Week4 vs. Week 6 follow-up	0 (0, 0)	–
Control group	Baseline vs. Week 2	0.00 (0.00, 0.00)	–
	Baseline vs. Week 4	0.00 (0.00, 0.00)	–
	Baseline vs. Week 6 follow-up	−0.09 (−0.25, 0.07)	0.828
	Week 2 vs. Week 4	0.00 (0.00, 0.00)	–
	Week 2 vs. Week 6 follow-up	−0.09 (−0.25, 0.07)	0.828
	Week4 vs. Week 6 follow-up	−0.09 (−0.25, 0.07)	0.828

Intergroup comparisons (Table 7) showed that differences between the two intervention groups and the control group became apparent from Week 2 onwards. By week 2, the PSQI score in the Binaural Beats combined with pure music group was already significantly lower than that of the control group, with a mean difference of −0.91 (95% CI: −1.70 to −0.12), adjusted *p* = 0.017; this difference was further confirmed at week 4 (mean difference −1.23, 95% CI: −1.98 to −0.48, *p* < 0.001) and at the 6-week follow-up (mean difference −1.32, 95% CI: −2.09 to −0.55, *p* < 0.001). The differences between the Pink Noise combined with pure music group and the control group at weeks 2 and 4 were not statistically significant (*p* = 0.228; *p* = 0.092), but a difference became apparent by week 6 (mean difference −0.86, 95% CI: −1.71 to −0.02, *p* = 0.044). There were no statistically significant differences in PSQI scores between the two intervention groups at any time point (*p* = 0.319–0.659).

Intra-group comparisons (Table 8) indicated that PSQI scores in the Pink Noise combined with pure music group decreased significantly following the intervention: at the 2-week, 4-week and 6-week follow-ups, they were significantly lower than baseline (mean differences of 0.41, 0.59 and 0.64, *p* ≤ 0.001), but no further significant changes were observed between time points after Week 2 (*p* = 0.162–1.000), suggesting that sleep quality in this group had largely improved by Week 2 and subsequently entered a plateau phase. The Binaural Beats combined with pure music group showed a greater degree of improvement and a progressive trend: a decrease of 0.86 from baseline by week 2 (95% CI: 0.67–1.06, *p* < 0.001), with the reduction from baseline increasing to 1.18 by weeks 4 and 6 (95% CI: 0.82–1.55, *p* < 0.001); Furthermore, the scores at weeks 4 and 6 were significantly lower than those at week 2 (mean difference 0.32, 95% CI: 0.06–0.58, *p* = 0.008), indicating that sleep quality in this group continued to improve during the intervention period and stabilized by week 4. No statistically significant differences were observed in pairwise comparisons between time points within the control group (*p* > 0.05), and the overall time effect was also not significant (Wald χ^2^ = 2.200, *p* = 0.138).

In summary, both music intervention protocols effectively improved the sleep quality of university students. The Binaural Beats combined with pure music group showed an earlier onset of effect (differences from the control group were observed as early as week 2), a greater degree of improvement, and a pattern of sustained and progressive improvement; the Pink Noise combined with pure music group exhibited a relatively smaller degree of improvement, with a significant gap from the control group only emerging by week 6. No meaningful fluctuations in sleep quality were observed in the control group throughout the study period.

## 4 Discussion

This randomized controlled trial, conducted within the naturalistic living environment of college students, evaluated the effects of two distinct audio protocols (both integrated with pure music) on participants’ sleep quality, perceived stress levels, and morning salivary cortisol—an objective biomarker reflecting stress system activity and sleep–wake rhythm (Backhaus et al., 2004; Begum et al., 2022). The core findings can be summarized as follows: In terms of subjective perception, both intervention programs showed significant effects, but they differed in terms of trajectory and specificity of improvement. In terms of pressure perception (PSS), the binaural beats group was significantly better than the control group and the pink noise group (mean difference of 2.55 points), and the improvement pattern was characterized by a “continuous progression-stabilization”; in contrast, the pink noise group was effective, but the magnitude of the improvement was relatively limited. In terms of sleep quality (PSQI), although both groups showed significant improvement from baseline, and the binaural beats group showed a greater decrease in PSQI (0.86 at week 2, 1.18 at weeks 4 and 6) than the pink noise group (0.41 to 0.64 at the same time), the statistical test showed that the difference in PSQI between the two groups did not reach the level of significance (*p* = 0.319–0.659), i.e., it was not possible to distinguish between the two types of sound intervention. That is, it is not yet possible to distinguish between the advantages and disadvantages of the two sound interventions.

The analysis of salivary cortisol revealed a more complex pattern among objective physiological indicators. Although the analyses revealed a significant main effect of time, indicating an overall change in cortisol levels throughout the study period, neither the between-group main effect nor the time-group interaction was significant. The significant improvement in subjective experience did not correspond to a clear and consistent pattern of between-group changes in morning cortisol levels, possibly because, first, the sound intervention’s limited characteristics meant the present study failed to completely shield subjects from the expectancy effect, although it achieved single-blindness of assessors. Subjects’ positive expectations arising from perceived participation in the sound intervention study may have amplified the magnitude of improvement in the subjective questionnaires to some extent. Second, there may be a more complex relationship between subjective physical and psychological benefits and the short-term modulation of secretory homeostasis underlying HPA axis activity. A variety of factors, such as the measurement time point, individual differences, and the time scale and pathway specificity of responses across physiological systems, may influence this complex association. Its specific mechanism needs to be further elucidated in future studies. However, given that the binaural beats group and the pink noise group were highly matched in terms of intervention context and psychological expectations, the two groups showed a significant between-group difference on the PSS, which, to a certain extent, excludes the confounding of psychological cues alone and confirms the specific value of the binaural beats intervention.

### 4.1 Mechanisms and differential analysis of subjective improvement

The consistent effects of binaural beats in improving sleep quality and relieving stress can be explained at several levels. At the theoretical level, its effects are mainly based on the brainwave synchronization hypothesis (Huang & Charyton, 2008). That is, auditory stimuli at specific frequencies can direct cortical electrical activity toward rhythms of external signals, thereby potentially modulating the EEG state toward frequency bands associated with relaxation and sleep (e.g., alpha, delta, theta waves) (Thatcher et al., 2009). At the physiological level, some studies have observed, with the help of electrocardiography, that binaural beats can modulate the autonomic nervous system, potentially inhibiting sympathetic activity, increasing parasympathetic tone, stabilizing the heart rate, decreasing the overall level of physiological arousal, and facilitating the body’s transition to a state of relaxation and sleep (Lee et al., 2024). Binaural beats may optimize functional connectivity in the prefrontal cortex and correct connectivity abnormalities and lateralization in stressful states to some extent, both of which provide a potential neural basis for their alleviation of subjective perceptual stress, as revealed by EEG (Al-Shargie et al., 2022; Scala et al., 2025). Thus, from possible EEG rhythm guidance, autonomic function modulation to prefrontal connectivity optimization, these mechanisms together form the physiological basis for binaural beats to improve sleep and stress perception (Garcia-Argibay et al., 2019; Ingendoh et al., 2023). The results of this study in real-life scenes further support these findings (Beauchene et al., 2016; Chockboondee et al., 2023; Jirakittayakorn & Wongsawat, 2018; McConnell et al., 2014; Perales et al., 2019; Picton et al., 2003; Ross et al., 2014; Tanaka & Kirino, 2019). In contrast, pink noise improves the sleep environment primarily through acoustic masking, which relies on shielding against environmental noise. It was found that pink noise did not improve overall sleep structure, and its improvement in sleep quality was relatively limited when individuals adapted to the acoustic environment compared with quiet conditions (Vincens et al., 2026). This may explain why the effects of the pink noise intervention stabilized after the initial presentation and were not significantly different from those of the control group in the later stages. In terms of regulating stress, the study found that, unlike ordinary noise, which alters the body’s metabolism and poses a disease risk, pink noise does not cause such changes and does not pose a disease risk (Vincens et al., 2026). Instead, it can have a moderating effect on attention, which is effective at reducing pain and stress (Araman et al., 2026). The difference in subjectively reported effects between the binaural beat group and the pink noise group further supports the finding that different acoustic properties work differently, suggesting that future applications need to be tailored to specific goals (short-term noise shielding vs. long-term neuromodulation) (Askarpour et al., 2024; Vincens et al., 2026).

### 4.2 Dissociation between subjective and objective measures

The present study found that salivary cortisol concentrations were not significantly affected by the intervention, consistent with findings from some studies. This separation may stem from the following points: first, cortisol is inherently complex as an indicator of assessment. Not only is it a stress hormone, but it is also involved in regulating daytime energy and arousal states, and there is a bidirectional, not unidirectional, association with sleep disorders. Studies have shown that chronic stress may lead to shorter sleep duration and lower morning salivary cortisol levels (Backhaus et al., 2004), while sleep deprivation or acute stress may also cause elevated morning salivary cortisol levels (Hernández et al., 2018). In addition, baseline cortisol levels varied significantly among individuals, meaning that even within the same “poor sleeper” group, baseline values may show different patterns of highs, lows, or increasing fluctuations, making static group-level measurements susceptible to masking individualized trends. Second, the measurement design of this study has its own practical limitations and corresponding characteristics. Due to limitations, this study did not collect salivary cortisol data at multiple time points within a day, thereby failing to capture cortisol arousal responses or complete circadian secretion dynamics, which are more sensitive to psychobehavioral interventions (Yang Z. et al., 2025). To obtain as much data as resources allowed, the present study utilized a longitudinal repeated-measures protocol, in which peak salivary cortisol was measured on four occasions at baseline, intervention, and follow-up, for a total of 30 min at morning awakening. However, the results of generalized estimating equation analysis showed that only one between-group difference was observed at week 4. This further suggests that a single static indicator is insufficiently stable and sensitive to capture changes in physiological indicators triggered by sound interventions. In studies using animal models, it has also been noted that physiological data collected at a single time point may obscure dynamic regulatory responses. (Zhang et al., 2024)Future studies that incorporate dynamic monitoring at multiple time points may help to reveal more clearly the characteristics and mechanisms of endocrine responses under different modes of sound intervention. Finally, this may be related to the specific pathways through which sound interventions exert their effects. Such interventions may relieve subjective stress and promote sleepiness primarily through rapid modulation of central nervous system excitability (e.g., by reducing sympathetic tone) (McConnell et al., 2014). Such immediate modulatory effects targeting neural states may not be directly and equally expressed in endocrine hormone levels. Such discordance has also been noted in studies of neurodegenerative disorders (Zhang et al., 2026). In summary, the findings of this study suggest that when evaluating non-pharmacological programs such as sound interventions, attention should be paid to the combination of subjective and objective indicators, and to dynamic monitoring at as many time points as possible when conditions permit, to reveal their mechanisms of action more comprehensively and sensitively. Meanwhile, under realistic research conditions, clarifying the limitations and strengths of the measurement design is also important for rationally interpreting the results and planning future studies.

### 4.3 Limitations and future directions

The limitations of this study are primarily evident in three areas: the depth of the measurement dimensions, the rigour of the statistical inferences, and the limitations of compliance monitoring methods and self-reporting.

First, the breadth and dynamics of the physiologic measurements were insufficient. To control the burden and cost of the study, only longitudinal morning cortisol measurements were used, which failed to capture sensitive intra-day dynamics (e.g., the cortisol awakening response) or indicators of synchronized neural activity. The single sample was susceptible to circadian rhythms and situational fluctuations, limiting in-depth elucidation of the underlying neuroendocrine mechanisms. Future studies should deepen the measurement by using area under the curve (AUC) analysis and multiple daily acquisitions, combined with autonomic indicators (e.g., HRV) or multimodal EEG technology, to build a more reliable neuroendocrine assessment system and accurately match the dynamic trajectory of the intervention.

Second, the potential interference of baseline extremes with mean reversion. The present study used a PSQI score> 7 as the inclusion criterion, which ensured the clinical relevance of the sample but may also introduce a risk of mean reversion. I.e., extremely high baseline scores show a decreasing trend in subsequent measurements due to the natural fading of random errors, thereby partially confounding the true effect of the intervention. Although this effect was somewhat stripped away in this study through strict randomization grouping (balancing baseline differences between groups) and modeling using generalized estimating equations (GEE), and the significantly larger declines in the two intervention groups than in the control group support the true validity of the intervention, this statistical bias is still not negligible. Future studies could use a double baseline design (Double Baseline) or incorporate baseline values as covariates in their analyses to more tightly control such bias.

Finally, although this study achieved a zero dropout rate, this may be attributed to a variety of contextual factors. Firstly, the study was conducted in a boarding school setting; the inclusion criteria required participants to have “no plans for extended absences” and to comply with daily dormitory checks, which to some extent improved the accessibility of follow-up. Secondly, the process of collecting salivary cortisol samples was relatively straightforward and was viewed by some participants as a low-burden procedure akin to a “health check-up”, which may have enhanced participation and compliance. Furthermore, the audio intervention utilized a hybrid format based on instrumental music; no significant feedback regarding auditory discomfort was received, which also helped to maintain sustained participation. Finally, this study adopted a payment method whereby participant fees were distributed in a single lump sum upon completion of the entire study process, rather than in installments. This design may have, to some extent, strengthened participants’ motivation to complete the full follow-up, thereby reducing the likelihood of voluntary withdrawal. It should be noted that, although the follow-up completion rate was high, the assessment of intervention adherence in this study relied primarily on participants’ self-reports, rather than objective verification via a device-based backend system. Consequently, the influence of social desirability bias (where participants may tend to overestimate their actual listening duration to meet study expectations or receive remuneration) and recall bias (reporting based on subjective memory rather than precise records) cannot be entirely ruled out. Future research may consider introducing objective monitoring methods based on smart devices or wearable technology to improve the accuracy of compliance data, and further validate the robustness of this study’s findings under conditions that more closely resemble natural living environments.

## 5 Conclusion

This study found that listening to pink noise or binaural beats, combined with pure music, before bedtime reduced stress levels and improved sleep quality among college students. Among the two interventions, the binaural beats protocol demonstrated more sustained and pronounced improvements in subjective outcomes over time. However, these perceived benefits were not yet accompanied by consistent between-group differences in morning salivary cortisol levels. This pattern may suggest that the effects of sound-based interventions are more readily reflected in subjective experiences of stress and relaxation (Chockboondee et al., 2023). At the same time, because cortisol was measured at a single daily time point, potential changes in endocrine regulation—particularly dynamic variations in hypothalamic–pituitary–adrenal axis activity—may not have been fully captured by the current measurement approach. Overall, these findings contribute to the growing literature on non-pharmacological sleep interventions and highlight the potential of binaural beats–based audio protocols as accessible tools for promoting sleep-related well-being among university students.

## 6 Institutional review board statement

This research protocol has been submitted to the Office of Scientific Research and International Affairs at Henan Forestry Vocational College for review and has been approved (Approval Date: November 6, 2025). This study employs a randomized controlled trial design to deliver a 4-week pre-sleep auditory intervention (pink noise and binaural beats) to college students and involves collecting salivary cortisol samples. The study strictly adheres to the Declaration of Helsinki and relevant international ethical guidelines. All experimental procedures were reviewed ethically prior to implementation to ensure they would not harm participants’ physical or mental health. All study participants are college students with full legal capacity; no vulnerable groups are involved. During data collection and processing, the research team strictly protects participant privacy; all data are coded and analyzed anonymously and used solely for scientific research purposes.

## 7 Informed consent/consent to participate

All participants signed a written informed consent form prior to the start of the study. The researchers explained in detail to the participants before the experiment the purpose of the study, the experimental procedure, the content of the sound intervention (including pink noise and binaural beats), the method of saliva sample collection, and possible minor discomforts (e.g., the discomfort of wearing headphones to sleep, etc.). Participants were explicitly informed that their participation was entirely voluntary and that they could withdraw unconditionally at any stage of the study without any adverse consequences. All participants confirmed that they had fully understood the study and agreed to participate. In addition, the study guarantees that all data will be used solely for academic research purposes and that participants’ personal information will be treated with strict confidentiality to ensure anonymity and data security.

## Data Availability

The raw data supporting the conclusions of this article will be made available by the authors, without undue reservation.
